# Integration of Patient-Reported Outcome Measures in Clinical Practice for Head and Neck Cancer Patients: A Cross-Sectional Survey

**DOI:** 10.3390/curroncol33050275

**Published:** 2026-05-08

**Authors:** Tatiana Dragan, Niclas Hubel, Jens Lehmann, Katherine J. Taylor, Renée Bultijnck, Tihana Gašpert, Luigi Lorini, Vincent Bourbonne, Arnaud Beddok, Bartłomiej Tomasik, Daan Nevens, Stefano Cavalieri, Ruth Gabriela Herrera Gómez, Esmée Lauren Looman, Iyizoba-Ebozue Zsuzsanna, Fatjona Kraja, Emma Lidington, Csongor György Lengyel, Marc Oliva, Gerardo Petruzzi, Ana Varges Gomes, Maria Pilar Solis Hernandez, Sophie Veldhuijzen van Zanten, Jesus Brenes Castro, Francesca Caparrotti, Giuseppe Fanetti, Yannick G. Eller, Chiara Gottardi, Laurelie R. Wishart, Petr Szturz

**Affiliations:** 1 Department of Radiation Oncology (Head and Neck Unit), Institut Jules Bordet, Hôpital Universitaire de Bruxelles (HUB), Université Libre de Bruxelles, 1180 Brussels, Belgium; 2 University Hospital of Psychiatry II, Medical University of Innsbruck, 6020 Innsbruck, Austria; niclas.hubel@i-med.ac.at (N.H.); jens.lehmann@tirol-kliniken.at (J.L.); 3 Department of Quality of Life, University Hospital Rostock, Comprehensive Cancer Center Mecklenburg-Vorpommern, 18057 Rostock, Germany; kataylor@uni-mainz.de; 4 Institute of Medical Biostatistics, Epidemiology, and Informatics, University Medical Centre Mainz, 55131 Mainz, Germany; 5 Department of Human Structure and Repair, Ghent University, 9000 Ghent, Belgium; renee.bultijnck@ugent.be; 6 Faculty of Health Sciences, University of Maribor, 2000 Maribor, Slovenia; tihana.gaspert@student.um.si; 7 University Hospital Rijeka, 51000 Rijeka, Croatia; 8 Medical Oncology and Hematology Unit, IRCCS Humanitas Research Hospital, 20089 Rozzano, Italy; luigi.lorini@cancercenter.humanitas.it; 9 Department of Radiation Oncology, Institut de Cancérologie et d’Imagerie, University Hospital, 29609 Brest, France; vincent.bourbonne@chu-brest.fr; 10 INSERM LaTIM, UMR 1101, Université de Bretagne Occidentale, 29200 Brest, France; 11 Department of Radiation Oncology, Institut Godinot, 51454 Reims, France; arnaud.beddok@reims.unicancer.fr; 12 CRESTIC, Université de Reims Champagne-Ardenne, 51000 Reims, France; 13 Department of Oncology and Radiotherapy, Medical University of Gdańsk, 80-210 Gdańsk, Poland; bartlomiej.tomasik@gumed.edu.pl; 14 Centre for Experimental Cardiooncology, Medical University of Gdańsk, 80-210 Gdańsk, Poland; 15 Radiotherapy Department, Kortrijk Cancer Centre, AZ Groeninge, 8500 Kortrijk, Belgium; daan.nevens@azgroeninge.be; 16 Faculty of Medicine and Health Sciences, University of Antwerp, 2610 Antwerp, Belgium; 17 Head and Neck Medical Oncology Department, Fondazione IRCCS Istituto Nazionale dei Tumori, 20133 Milan, Italy; stefano.cavalieri@istitutotumori.mi.it; 18 Department of Oncology and Hemato-Oncology, University of Milan, 20122 Milan, Italy; 19 Department of Oncology, Hôpital Cantonal de Fribourg, 1708 Fribourg, Switzerland; ruthgabriela.herreragomez@h-fr.ch; 20 Department of Radiation Oncology, University Hospital Zurich, 8952 Zurich, Switzerland; esmee.looman@usz.ch; 21 National Health Service (NHS), London W2 1NY, UK; zsuzsanna.iyizoba@nhs.net; 22 Surgery Department, Faculty of Medicine, University of Medicine Tirana, 1005 Tirana, Albania; fatjona.kraja@umed.edu.al; 23 University Hospital Center Mother Teresa, 1005 Tirana, Albania; 24 Centre for Cancer Screening, Prevention and Early Diagnosis, Queen Mary University of London, London E1 4NS, UK; e.lidington@qmul.ac.uk; 25 Department of Head and Neck Surgery, National Institute of Oncology, 1122 Budapest, Hungary; lengyel.csongor@gmail.com; 26 Medical Oncology Department, Institut Català d’Oncologia (ICO), L’Hospitalet de Llobregat, 08908 Barcelona, Spain; moliva@iconcologia.net (M.O.); jesusbrenescastro@gmail.com (J.B.C.); 27 Institut d’Investigació Biomèdica de Bellvitge (IDIBELL), 08908 Barcelona, Spain; 28 Otolaryngology and Head and Neck Surgery, IRCCS Regina Elena National Cancer Institute, 00144 Rome, Italy; gerardo.petruzzi@ifo.it; 29 Medical Oncology Department, Unidade Local de Saúde do Algarve, 8500-338 Portimão, Portugal; ana.varges.gomes@chalgarve.min-saude.pt; 30 Medical Oncology Service, Hospital Universitario Central de Asturias, 33011 Oviedo, Spain; mariadelpilar.solis@sespa.es; 31 Department of Radiology and Nuclear Medicine, Erasmus MC, 3015 GD Rotterdam, The Netherlands; s.veldhuijzenvanzanten@erasmusmc.nl; 32 Erasmus MC Cancer Institute, 3015 CP Rotterdam, The Netherlands; 33 Department of Radiation Oncology, Clinique Générale Beaulieu, Swiss Medical Network, 1206 Geneva, Switzerland; fcaparrotti@beaulieu.ch; 34 Division of Radiation Oncology, Centro di Riferimento Oncologico di Aviano (CRO) IRCCS, 33081 Aviano, Italy; giuseppe.fanetti@cro.it; 35 Centre for Medical Education, University of Dundee, Dundee DD1 4HN, UK; 2421684@dundee.ac.uk; 36 Istituto Oncologico Veneto (IRCCS), 35128 Padova, Italy; chiara.gottardi@iov.veneto.it; 37 School of Medicine and Dentistry, Griffith University, Brisbane, QLD 4111, Australia; laurelie.wishart@health.qld.gov.au; 38 Office of the Chief Allied Health Practitioner, Metro North Hospital and Health Service, Brisbane, QLD 4029, Australia; 39 School of Health and Rehabilitation Sciences, The University of Queensland, Brisbane, QLD 4072, Australia; 40 Department of Oncology, University of Lausanne and Lausanne University Hospital, 1011 Lausanne, Switzerland; szturz@gmail.com

**Keywords:** PROM implementation, head and neck cancer, usage patterns, perceived value, implementation barriers

## Abstract

**Background:** Head and neck cancer (HNC) and its multimodal treatment substantially impair speech, swallowing, breathing, appearance, and psychosocial well-being. Patient-reported outcome measures (PROMs) improve symptom monitoring and quality of life in oncology, yet their integration into routine HNC care remains inconsistent. This study assessed patterns of PROM use, perceived value, and barriers to implementation among healthcare professionals (HCPs) involved in HNC care. **Methods:** A 30-item cross-sectional survey was distributed to HCPs treating HNC patients between June 2024 and April 2025. The questionnaire explored PROM use in clinical practice and trials, perceived relevance across care phases, and implementation barriers. Respondents were classified as non-users, occasional users, or regular users. Data were analyzed descriptively with comparisons between groups. **Results:** Among 133 respondents, 33.8% were non-users, 29.3% occasional users, and 36.8% regular users of PROMs. Users reported inviting half of patients to complete PROMs, predominantly via paper-based questionnaires (67.8%). PROMs were mainly applied during active treatment and early follow-up to monitor symptoms, overall health, and emotional well-being, and were less frequently used to guide treatment decisions. The EORTC QLQ-C30 and HNC-specific tools were most commonly reported. Compared with users, non-users more often cited lack of time, limited training in interpreting PROM data, insufficient institutional support, resource constraints, and lack of appropriate instruments (all *p* < 0.05). PROM use in clinical trials was associated with routine use (*p* < 0.001). **Conclusions:** Although PROMs are widely valued in HNC care, their integration into clinical decision-making remains limited. Addressing organizational, educational, and digital barriers is essential to support sustainable implementation.

## Simple Summary

Head and neck cancer and its treatments can greatly affect patients’ ability to speak, swallow, breathe, and maintain social interactions, leading to a significant impact on daily life. Questionnaires that allow patients to report their own symptoms and well-being are increasingly used in cancer care because they help clinicians better understand patients’ needs. However, their routine use in head and neck cancer care is still inconsistent. In this study, we surveyed healthcare professionals to understand how often these tools are used, how useful they are perceived to be, and what challenges limit their implementation. Although most professionals recognize their importance, these tools are mainly used to monitor symptoms rather than to guide treatment decisions. Common barriers include lack of time, training, and institutional support. Addressing these challenges could improve patient-centered care and support better integration into everyday clinical practice.

## 1. Introduction

Head and neck cancer (HNC) presents unique clinical challenges due to its anatomical complexity and the vital functions affected by the disease and its treatment. The management of HNC typically requires multi-modal therapeutic strategies, including surgery, radiation therapy, chemotherapy, and increasingly, immunotherapy with parallel integration of supportive care services. Each of these modalities, alone or in combination, can profoundly affect patients’ health-related quality of life (HRQOL) by impacting critical functions such as speech, swallowing, breathing, and appearance [1,2]. The aggressive nature of these treatments often leads to significant physical, psychological, and social impact burdens, making the assessment of functional outcomes and patient well-being essential components of comprehensive care. Patient-reported outcome measures (PROMs) capture patients’ perspectives on their symptoms and HRQOL through validated questionnaires. Originally developed within the context of clinical trials and research [3], PROMs are increasingly finding application in routine clinical practice [4]. Randomized controlled trials have consistently shown that routine symptom monitoring via PROMs improves clinical outcomes, optimizes patient experience, and reduces healthcare utilization [5,6,7]. Recently, a meta-analysis demonstrated that PROM interventions incorporating tailored feedback and clinician alerts enhance effectiveness in care delivery, with observed improvements in HRQOL and potential survival benefits [8,9]. Specifically, PROM-based interventions have been associated with long-term HRQOL gains in lung cancer patients [6,7], significant reductions in symptom burden [10], and enhanced safety and quality of care in patients receiving immunotherapy while reducing clinician monitoring time [11].

Taken together, these findings support the view of PROMs as a paradigm shift in oncology care, although translation of trial-based benefits into routine HNC practice remains variable [12]. Supported by growing clinical evidence [13,14], several oncological societies, including the European Society for Medical Oncology (ESMO), recommend using PROMs in cancer care [15,16]. Despite the increasing interest in and use of PROMs for patients with HNC [17,18], their routine implementation remains limited. Barriers to adoption may include logistical challenges, limited digital infrastructure, time constraints, uncertainty regarding the interpretation of PROM data, and insufficient training or awareness among healthcare professionals (HCPs) [19,20,21].

Previously, we conducted a cross-sectional survey among HCPs within the European Organisation for Research and Treatment of Cancer (EORTC) focusing on the use of PROMs in routine clinical practice; among respondents: 43% reported not using PROMs, 27% used them occasionally, and only 30% reported regular use [22]. Notably, HCPs treating HNC patients were among the least frequent users. In the present study, we focused exclusively on HNC and surveyed a broader community of HCPs to better understand their perspectives on PROMs and identify opportunities to improve their integration into the routine treatment of HNC patients. The major topics addressed in the survey were the value of PROMs, the current patterns of use, and the preferences regarding optimal approaches for PROM collection.

## 2. Materials and Methods

We conducted a cross-sectional survey across HNC HCPs. This study was reviewed and approved by the Ethics Committee of the Institute Jules Bordet in Brussels (approval number: 3749, approval date: 11 January 2024) and is reported here according to the Strengthening the Reporting of Observational Studies in Epidemiology (STROBE) guidelines [23].

### 2.1. Survey Development

The survey development was guided by a review of the literature on PROM implementation in oncology, focusing on facilitators, barriers, and clinical applications [24,25,26,27,28]. An initial pool of items in English was generated through collaboration between members of the Young and Early Career Investigator communities of the EORTC HNC and QOL groups, including surgeons, oncologists, radiation therapists, nurses, supportive care specialists, and patient-reported outcomes researchers. The first 25 questions of the survey were identical to those used in a previously conducted EORTC cross-tumor survey and were not specific to any single pathology [22]. Building on this, five additional questions specific to HNC were developed to address disease-specific considerations. The resulting 30-item survey underwent internal validation and pilot testing prior to dissemination. The draft version was pilot-tested among eight HCPs involved in HNC care to ensure clarity, face validity, and content relevance. The questionnaire comprised 30 items organized into five sections. The demographic section (10 questions) collected participant characteristics, including country of practice, professional role, years of experience in HNC, and type of healthcare facility. The clinical practice section (12 questions) looked at whether and how PROMs were used outside of clinical trials, including how often they were used, how they were given to patients, and what challenges non-users faced (like not having enough time, not getting support from their organization, or not having enough training). The implementation and practical aspects section (6 questions) addressed operational issues among PROM users, such as duration and timing of PROM use, staff responsibilities, patient adherence strategies, participation rates, and PROM instruments used (e.g., EORTC QLQ-C30, QLQ-H&N43, FACT-H&N, PROMIS) and their purposes. The clinical trial section (3 questions) evaluated experience with PROMs in research settings and preferences for future use. Finally, the HNC patient care section (5 questions) investigated how important PROMs are in regular care, what key areas should be evaluated, how familiar people are with HNC-specific tools, and their views on the evidence supporting these tools, ending with a question about how to better include PROMs in HNC.

The questionnaire comprised both closed-ended and open-ended items to collect quantitative and qualitative data. Closed-ended questions mainly used multiple-choice and Likert-type scales (like Never/Rarely/Sometimes/Often/Always) to help analyze the data, while open-ended questions gathered detailed information about participants’ views.

The complete survey is shown in the Supplementary Materials.

### 2.2. Data Collection

The survey was created using Survey Monkey (SurveyMonkey Inc., San Mateo, CA, USA). The EORTC distributed the survey via targeted email invitations and an open weblink to the Young and Early Career Investigator communities of the EORTC HNC Group and QOL Group, who further shared it within their institutions. Any HCPs involved in HNC patients regardless of current PROM use were invited to complete the survey. Examples of HCPs include HNC surgeons (otolaryngologists and maxillofacial), radiation oncologists, medical oncologists, nurses, speech therapists, dietitians, and psychologists. The survey was open between 17 June 2024 and 30 April 2025, with three emailed reminders during this period. Participation was voluntary and anonymous, and email addresses, collected solely for research correspondence, were kept confidential and unlinked to survey responses.

### 2.3. Data Analysis

Descriptive statistics were used to summarize respondents’ sociodemographic and professional characteristics. Participants were classified into three groups based on their self-reported use of PROMs in routine practice: non-users (never use PROMs); occasional users (use PROMs infrequently or on an occasional basis); and regular users (use PROMs at least monthly), with reported use ranging from a couple of times per month to almost daily. Group distributions and duration of PROM use were described, with exploratory comparisons by institutional setting (academic vs. non-academic). Methods of PROM use (questions 12–21), including mode and timing of administration, responsible HCP, PROM instruments used, and purposes of use, were analyzed descriptively. Likert-scale responses were treated as ordinal data and presented as frequency distributions or heat maps. Perceived usefulness of PROMs, relevant assessment domains, and preferred time points were summarized and compared between users and non-users. Barriers to PROM implementation were analyzed separately for users and non-users. Use of PROMs in clinical trials was summarized descriptively, and exploratory analyses examined associations with routine clinical use. Responses to the open-ended question were analyzed using descriptive content analysis to summarize participant perspectives. Group comparisons were primarily descriptive. When inferential testing was performed, statistical tests were selected according to variable type and number of compared groups, including independent-sample *t* tests and Kruskal–Wallis rank sum tests for continuous variables and chi-squared tests for categorical variables, using a two-sided significance level of 0.05. All analyses were conducted using R software (version 4.4.1) [29].

## 3. Results

A total of 600 email invitations were sent, resulting in 79 responses (response rate: 13.17%). Additionally, 90 responses were obtained via an open weblink; however, as this link could be freely forwarded, a response rate could not be calculated for this distribution channel. Overall, 169 individuals initiated the survey. Of these, 133 provided responses and were included in the analysis, while 36 did not answer any questions and were excluded.

Table 1 summarizes the sociodemographic and professional characteristics of all survey respondents (N = 133), as well as those stratified by their PROM use status: non-users (*n* = 45, 33.8%), occasional users (*n* = 39, 29.3%), and regular users (*n* = 49, 36.8%). Most respondents were based in Southern Europe (49%) or Western Europe (44%), with smaller proportions from Central and Eastern Europe (4%) or non-European countries (3.0%). A comprehensive list of countries is reported in the Supplementary Materials. Overall, respondents were predominantly physicians (85.0%), and women accounted for 51.9% of the sample.

Most participants reported substantial experience in HNC patient care, with 63.1% having more than 10 years of experience. The majority (72.9%) worked in academic or university hospitals and saw more than five new HNC patients each month. On average, respondents spent approximately half of their professional time in direct patient care (mean ± SD: 51.6 ± 25.7%). Across PROM user groups, distributions of gender, age, profession, years of experience, and type of workplace were broadly comparable.

### 3.1. Integration of PROMS in Clinical Practice

Among PROM users, either occasional or regular respondents (*n* = 88) reported that, on average, 50.5% (SD 35.0) of their patients were invited to complete PROMs at least once. PROMs were most commonly administered using paper questionnaires (67.8%), while 12.6% reported exclusive electronic administration and 19.5% reported using both formats. PROM completion most frequently occurred on-site at the healthcare institution (55.6%), although 34.6% of respondents indicated that PROMs were completed both at the institution and remotely at home.

PROMs were most often introduced to patients by physicians (62.2%) or nurses (34.1%). Approximately one-third of respondents reported PROM use within their own department only (33.0%), while 22.7% indicated use both within and outside their department; 29.5% were not aware of PROM use by other healthcare professionals at their institution. To support remote participation, respondents most frequently reported follow-up of non-adherent patients (40.0%) and patient training on questionnaire completion (37.1%), although 11.4% reported using no specific strategies. PROMs were most commonly used during active treatment (65.2%), followed by early follow-up (58.0%) and at diagnosis (55.1%). The most frequently used PROM instrument was the EORTC Core Quality of Life Questionnaire (EORTC QLQ-C30, 62.3%), followed by EORTC disease-specific questionnaires (36.2%) and other generic or symptom-focused instruments. More detailed results are presented in Table 2.

**Figure 1:**
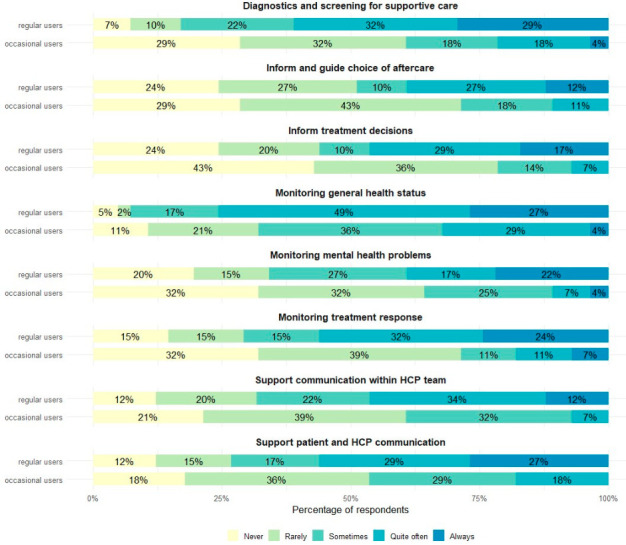
Reasons for using patient-reported outcome measures in clinical practice among regular and occasional PROM users (*n* = 88).

Figure 1 shows the reasons for using PROMs in clinical practice among regular and occasional PROM users. In both groups, the most common reasons for PROM use were monitoring general health status, supporting communication between patients and healthcare professionals, and monitoring mental health problems. Overall, PROMs were less frequently used to inform treatment decisions than for monitoring and communication purposes, particularly among occasional users.

### 3.2. Perceived Relevance and Value of PROMs in HNC Care

Most respondents reported that symptoms were assessed “always” or “quite often” (97.1%), followed by assessing emotional well-being (87.6%) and social functioning (85.7%); financial burden was less frequently assessed (52.4%). More than half of respondents (56.7%) believed there is substantial evidence supporting the clinical benefit and cost effectiveness of PROMs in HNC care, while 37.3% were unsure. PROMs were considered most valuable during active treatment (78.8%), early follow-up (71.2%), and at diagnosis (65.4%). The most frequently reported HNC-specific PROM was the EORTC QLQ-H&N43 (89.8%), followed by the MD Anderson Dysphagia Inventory (MDADI, 52.0%) and Functional Assessment of Cancer Therapy head and neck module (FACT-H&N, 40.8%).

Table 3 summarizes respondents’ perceptions of the relevance of PROM domains, the perceived evidence supporting PROM use, the phases of care in which PROMs are most valuable, and the PROM instruments considered relevant in HNC care.

### 3.3. Perceived Barriers for PROM Integration into Clinical Practice

Compared with PROM users (n = 88), non-users (n = 45) more frequently reported a lack of training in interpreting PROM results (62.2% vs. 35.2%, *p* = 0.005), lack of time (75.6% vs. 54.5%, *p* = 0.030), lack of support for implementation and use (75.6% vs. 45.5%, *p* = 0.002), limited institutional resources or infrastructure (64.4% vs. 43.2%, *p* = 0.033), and lack of appropriate PROMs (35.6% vs. 18.2%, *p* = 0.045). In contrast, accessibility concerns were reported more often by PROM users than non-users (48.9% vs. 28.9%, *p* = 0.043). No significant differences were observed for other perceived barriers (*p* > 0.05).

More detailed results are presented in Table 4.

Free text responses to Survey Question 33, “Based on your experiences, what suggestions do you have for improving the integration and utilization of PROMs in HNC patients’ clinical care practice?” were analyzed using qualitative descriptive methods. Among the 73 respondents who provided free text answers, the most frequently reported suggestions related to the need for improved infrastructure, time allocation, and dedicated human resources to support PROMs implementation. A large proportion of participants reported limited time during routine consultations and insufficient staffing as major barriers. Electronic collection of PROMs was commonly suggested, including integration into electronic health records and remote completion by patients at home via websites, mobile applications, tablets, or QR codes. Many respondents also emphasized the need for education and training of healthcare professionals to improve awareness, understanding, and interpretation of PROM data. Simplification of PROMs, including shorter questionnaires and improved ease of use, was frequently reported. Several participants highlighted the need for routine and standardized administration of PROMs across the care pathway, including during active treatment and follow-up, and their integration into clinical decision-making. Additional suggestions included the involvement of multidisciplinary teams in reviewing PROM data, clearer definitions of clinically meaningful changes, improved evidence supporting PROM use, and institutional, regulatory, or national-level support to facilitate broader implementation.

### 3.4. PROMs in HNC Clinical Trials

Overall, 68.8% of respondents reported having used PROMs in HNC clinical trials, including 23.9% who reported using PROMs all the time in clinical trials and 21.1% quite often. In contrast, 31.2% indicated that they had never used PROMs in a trial setting. Among those with experience using PROMs in trials, administration was most commonly paper-based or mixed paper–electronic (both 48%), while exclusive electronic administration was uncommon (4%). When asked about preferred administration in a hypothetical clinical trial, most respondents favored electronic-only (44%) or mixed-format (38.5%) approaches, with fewer preferring paper-only administration (17.4%). PROM use in clinical trials was correlated with PROM use in clinical practice (χ^2^(1) = 24.982, φ = 0.479, *p* < 0.001).

## 4. Discussion

This study offers context for current patterns of PROM use in HNC care as perceived by HCPs. More than two-thirds of respondents reported using PROMs at least occasionally in clinical practice, with over one-third identifying as regular users. These results add to the growing body of research that shows how oncologists are increasingly using PROMs in their work [19,20,21,22].

The amount of PROM use found in this study is similar to or slightly higher than what earlier oncology surveys showed, where about 50–70% of HCPs reported using some form of PROM and 30–45% said they do not use them regularly, depending on the type of cancer, location, and clinical setting [22,25]. Paper-based questionnaires, alone or combined with electronic formats, remain the predominant mode of PROM administration, despite growing evidence in support of ePROM systems [4]. The feasibility of ePROM implementation in HNC care has been demonstrated in routine practice and clinical trials. Using the “OncoFunction system”, Zebralla et al. reported high completion rates (78.5% of follow-up visits) and improved efficiency over time, supporting the practicality of electronic PROM collection during aftercare while also highlighting the need to address digital accessibility to ensure equitable use [30]. Similarly, in the randomized APCOT trial including 100 patients with HNC, Sprave et al. showed that app-based ePRO monitoring during radiotherapy was highly feasible, with 100% adherence to predefined criteria, and was associated with improved patient satisfaction despite comparable overall QOL outcomes relative to standard care [31]. The limited use of exclusively electronic PROMs in routine practice likely reflects infrastructural, organizational, and resource-related constraints.

In our survey, PROMs were most commonly used to monitor general health status, support patient–clinician communication, and assess mental health. In contrast, their use to inform treatment decisions was less frequent, particularly among occasional users, although PROMs could, for example, initiate referral for swallowing and or voice rehabilitation, nutritional interventions, escalation to additional diagnostic evaluation including imaging, or treatment adaptation, beyond their use for monitoring and communication purposes between patients and clinicians. This pattern is consistent with evidence showing that PROMs are primarily applied for symptom monitoring and supportive care, while their integration into clinical decision-making remains limited in routine oncology and HNC practice [32,33]. Interestingly, although PROMs are most frequently used to monitor symptoms and health status, their use remains largely confined to in-person hospital visits rather than remote or continuous assessment. In the context of HNC, where symptom trajectories can change rapidly during (chemo) radiotherapy and survivorship, more continuous PROM monitoring may help identify clinically meaningful deterioration earlier and support timely clinical evaluation, including targeted diagnostic work-up and treatment adjustment when appropriate. As a result, the full potential of PROMs, especially for finding worsening symptoms early and acting quickly, may not be fully realized. To improve ongoing monitoring in cancer care, it would be helpful to use electronic patient-reported outcome measures (ePROMs) more systematically and include them in regular clinical processes.

Symptoms, emotional well-being, and social functioning were identified as priority domains for PROM assessment, consistent with evidence demonstrating persistent functional and psychosocial impairments after HNC treatment that are not fully captured by traditional clinical outcomes [34]. Financial burden was less frequently prioritized, in line with reports of variable integration of financial toxicity into routine outcome assessment [35]. This may partly reflect geographic variation in PROM utilization, as respondents practicing in high-income settings or healthcare systems with broader coverage and social support infrastructure may perceive financial toxicity as less salient, influencing its inclusion in outcome measurement. More than half of respondents reported that substantial evidence supports the clinical benefit and cost effectiveness of PROMs, while over one-third reported uncertainty, reflecting previously described variability in clinician awareness of PROM-related evidence [32].

The PROM instruments most frequently reported, EORTC QLQ-H&N43, MD Anderson Dysphagia Inventory, and FACT-H&N, align with systematic review recommendations supporting EORTC QLQ-H&N43, QLQ-C30, and QLQ-OH15 for health-related QOL assessment, and identifying the “Swallowing Outcome After Laryngectomy” questionnaire as the only dysphagia-specific PROM meeting COnsensus-based Standards for the selection of health Measurement INstruments (COSMIN standards) [36,37].

Our survey identified clear differences in perceived barriers between PROM users and non-users, with non-users more frequently reporting lack of training in interpreting PROM results, lack of time, insufficient support for implementation, limited institutional resources or infrastructure, and lack of appropriate PROMs. These findings suggest that non-use of PROMs in HNC care is largely associated with organizational and capacity-related constraints rather than a lack of perceived clinical value. These results match what Nguyen et al. found, showing that healthcare professionals working in HNC care often see limited awareness and knowledge of PROMs, not enough time, and poor infrastructure as obstacles to regularly using PROMs [19].

Further external validation is provided by a recent large cross-tumor survey conducted among 784 EORTC healthcare professionals, which identified lack of time and insufficient support as the most frequently reported barriers to PROM use and showed that PROM users more often reported patient-level barriers, such as accessibility concerns [22]. Notably, the first 25 items of our survey were identical to those used in the EORTC study, enabling direct comparison across cancer types, while additional HNC-specific items extended the instrument to capture disease-specific considerations. The close alignment of findings across both surveys supports the robustness and generalizability of the observed implementation patterns.

A systematic review of 83 studies showed that successful integration of patient-reported outcomes in routine oncology practice depends on strong institutional support, clear workflows, and continuous training of healthcare teams, and that PROM use improves communication, identification of unmet needs, symptom monitoring, and cost effectiveness assessment [38]. These findings support the view of PROMs as a paradigm shift in oncology care and reinforce the need to address organizational and educational barriers to enable sustainable PROM implementation. Similarly, a systematic review by Foster et al. emphasized the importance of early investment in PROM strategy design, clinician preparation, electronic system development, and clarity regarding clinical use of PROM data, with lack of time, resources, and organizational readiness consistently identified as key barriers [20]. Together, these findings reinforce the need to address organizational and educational factors to enable sustainable PROM implementation in HNC care.

Most respondents reported experience using PROMs in HNC clinical trials, with nearly half indicating frequent or routine use. This finding is consistent with the established role of PROMs as key endpoints in oncology trials, particularly in HNC, where QOL outcomes are central to treatment evaluation. The choice of using electronic or a mix of electronic and paper methods for PROMs in imagined trials shows that digital PROM platforms are becoming more important in research.

This study is among the few surveys focusing specifically on PROM use in HNC care across multiple European settings with some international representation.

Several limitations should be acknowledged. First, the number of respondents (n = 133) is relatively small compared with the large number of healthcare professionals involved in the care of head and neck cancer patients across Europe. Participation was voluntary, which may have introduced selection bias, with respondents potentially being more interested in or familiar with PROMs than the broader HCP population. Therefore, the findings should be interpreted with caution and considered exploratory rather than representative of standard care across all countries and healthcare settings. In addition, the predominance of respondents from academic settings and physician roles may limit generalizability to other professional groups and care environments. PROM use may therefore be overestimated compared with non-academic settings, although no significant differences by institution type were observed. Furthermore, dependence on self-reported data may result in reporting bias. Finally, while barriers to PROM use were explored, facilitators were not assessed in detail; future studies should adopt implementation science frameworks to identify both barriers and enablers.

## 5. Conclusions

This study demonstrates that PROMs are widely used in HNC care, particularly for symptom monitoring and communication, but are less consistently integrated into treatment decision-making. Non-users more often reported barriers related to training, time, institutional support, resources, and availability of PROMs, whereas users more frequently identified patient accessibility challenges. These barriers are largely consistent with those reported in general oncology settings, suggesting that longstanding implementation challenges persist in HNC care. Given the high and dynamic symptom burden associated with HNC, targeted implementation strategies adapted to the HNC context are needed, with particular emphasis on integrating electronic PROMs into clinical workflows and establishing clear escalation pathways, including supportive care referral and diagnostic or treatment evaluation, to translate PROM data into actionable care.

## Figures and Tables

**Table 1:** Respondent characteristics.

	Overall (N = 133)	Non-Users ^1^ (*n* = 45)	Occasional Users ^1^ (*n* = 39)	Regular Users ^1^ (*n* = 49)	*p * ^5^
Country of work ^2^ (*n*, column%)					0.387
Southern Europe	65 (48.9)	20 (44.4)	23 (59.0)	22 (44.9)	
Western Europe	59 (44.4)	22 (48.9)	12 (30.8)	25 (51.0)	
Central and eastern Europe	5 (3.8)	1 (2.2)	2 (5.1)	2 (4.1)	
Non-European	4 (3.0)	2 (4.4)	2 (5.1)	0 (0.0)	
Gender (*n*, column%)					0.515
Woman	69 (51.9)	19 (42.2)	22 (56.4)	28 (57.1)	
Man	62 (46.6)	25 (55.6)	17 (43.6)	20 (40.8)	
I use a different term	2 (1.5)	1 (2.2)	0 (0.0)	1 (2.0)	
Age group in years (*n*, column%)					0.153
18–29	6 (4.5)	5 (11.1)	1 (2.6)	0 (0.0)	
30–39	30 (22.6)	8 (17.8)	10 (25.6)	12 (24.5)	
40–49	48 (36.1)	14 (31.1)	17 (43.6)	17 (34.7)	
50–59	36 (27.1)	11 (24.4)	9 (23.1)	16 (32.7)	
60–69	13 (9.8)	7 (15.6)	2 (5.1)	4 (8.2)	
Profession (*n*, column%)					0.259
Physician (any medical specialty)	113 (85.0)	40 (88.9)	34 (87.2)	39 (79.6)	
Nurse	9 (6.8)	1 (2.2)	4 (10.3)	4 (8.2)	
Other ^3^	11 (8.3)	4 (8.9)	1 (2.6)	6 (12.3)	
Member of a medical society (*n*, column%)	67 (50.4)	28 (62.2)	15 (38.5)	24 (49.0)	0.092
External recruitment (*n*, column%)	82 (61.7)	31 (68.9)	28 (71.8)	23 (46.9)	0.028
Time working with HNC (*n*, column%)					0.159
Less than one year	2 (1.5)	2 (4.4)	0 (0.0)	0 (0.0)	
1–3 years	11 (8.3)	5 (11.1)	5 (12.8)	1 (2.0)	
4–10 years	36 (27.1)	7 (15.6)	12 (30.8)	17 (34.7)	
11–20 years	49 (36.8)	18 (40.0)	12 (30.8)	19 (38.8)	
More than 20 years	35 (26.3)	13 (28.9)	10 (25.6)	12 (24.5)	
Place(s) of work (multiple answers possible; *n*, column%)					
Academic/university hospital	97 (72.9)	36 (80.0)	24 (61.5)	37 (75.5)	0.145
Non-academic center ^4^	46 (34.6)	12 (26.7)	17 (43.6)	17 (34.7)	0.266
Number of new HNC patients per month (*n*, column%)					0.370
1–5	26 (19.5)	9 (20.0)	8 (20.5)	9 (18.4)	
6–15	53 (39.8)	18 (40.0)	11 (28.2)	24 (49.0)	
>15	54 (40.6)	18 (40.0)	20 (51.3)	16 (32.7)	
Percentage of work spent seeing patients (mean (SD))	51.60 (25.67)	56.69 (23.93)	48.41 (27.48)	49.47 (25.54)	0.271
Time using PROMs in clinical practice (*n*, column%)					0.660
Less than 1 year	9 (11.0)	0 (0.0)	6 (17.1)	3 (6.5)	
1–3 years	33 (40.2)	1 (100.0)	13 (37.1)	19 (41.3)	
4–5 years	17 (20.7)	0 (0.0)	7 (20.0)	10 (21.7)	
6–10 years	11 (13.4)	0 (0.0)	6 (17.1)	5 (10.9)	
More than 10 years	12 (14.6)	0 (0.0)	3 (8.6)	9 (19.6)	

^1^ Definitions: Non-users (never use PROMs); occasional users (occasionally or infrequently use PROMs); regular users (use PROMs at least monthly, up to daily). ^2^ Definitions according to EuroVoC (https://op.europa.eu/en/web/eu-vocabularies/concept-scheme/-/resource?uri=http://eurovoc.europa.eu/100277, accessed on 5 May 2026) ^3^ Supportive care professional (physiotherapist, nutritionist, advanced practitioner therapeutic radiographer, dentist, radiation therapist. ^4^ Including: regional hospital, serving a geographic region; county hospital, acute with less than 200 beds; tertiary referral hospital; private practice; ambulatory care; research institute ^5^ *p*-values based on chi-squared tests and Kruskal–Wallis rank sum test. Note: missing values not included in calculation of percentages.

**Table 2:** Current usage of PROMs in clinical practice (users only, n = 88).

	Users (*n* = 88)
Percentage of patients invited to complete PROMs at least once (mean (SD))	50.51 (34.99)
Mode of PROM assessment in clinical practice (*n*, column%)	
Paper questionnaires	59 (67.8)
Electronic questionnaire assessment	11 (12.6)
Both formats	17 (19.5)
Location of PROM completion (*n*, column%)	
On site at the institution	45 (55.6)
Remotely at home	8 (9.9)
Both	28 (34.6)
Who introduces PROMs to patients? (multiple answers possible; *n*, column%)	
Physicians	51 (62.2)
Nurses	28 (34.1)
Administrative staff	8 (9.8)
PRO coordinator (i.e., a person dedicated to PROM assessment)	6 (7.3)
I do not know	2 (2.4)
Other	5 (6.1)
Are other HCPs at your institution using PROMs? (*n*, column%)	
Yes, people at my department	29 (33.0)
Yes, people at other departments	13 (14.8)
Yes, people both at my department and other departments	20 (22.7)
No, not that I am aware of	26 (29.5)
Methods for ensuring remote participation (multiple answers possible; *n*, column%)	
Calling/following up non-adherent patients	14 (40.0)
Training patients on when and how to complete questionnaires	13 (37.1)
Optimizing the timing of assessments	10 (28.6)
Automated reminders (SMS, email, app)	9 (25.7)
Offering a support hotline or contact information	8 (22.9)
We do not use any strategies to ensure participation	4 (11.4)
Time of use in the care pathway (multiple answers possible; *n*, column%)	
During active treatment	45 (65.2)
During the early stages of follow-up (<23 years after end of treatment)	40 (58)
At diagnosis	38 (55.1)
During long-term follow-up (>23 years after end of treatment)	22 (31.9)
At disease progression or recurrence	15 (21.7)
During palliative care (after completion of anti-cancer therapy)	9 (13)
Other ^1^	1 (1.4)
PROMs used (multiple answers possible; *n*, column%)	
EORTC QLQ-C30	43 (62.3)
EORTC disease-specific questionnaires ^2^	25 (36.2)
FACIT questionnaires ^3^	12 (17.4)
HADS (Hospital Anxiety and Depression Scale)	12 (17.4)
Self-developed questionnaire	12 (17.4)
EQ-5D-5L	11 (15.9)
SF-36 ^4^	8 (11.6)
PRO-CTCAE (Patient Reported Outcomes version of the Common Terminology Criteria for Adverse Events)	7 (10.1)
Other EORTC questionnaires ^5^	4 (5.8)
Other	18 (26.0)

^1^ Radiotherapy Late Effects Clinic. ^2^ EORTC QLQ-BR23, EORTC QLQ-LC13. ^3^ Functional Assessment of Chronic Illness Therapy like the FACTG. ^4^ 36-item Short Form Health Survey or SF-12, Short Form Health Survey. ^5^ EORTC QLQ-PATSAT-C33, EORTC QLQ-COMU26.

**Table 3:** Perceived relevance, evidence, care phases, and PROM instruments for PROM use in HNC care (N = 133).

	N (%)
In your opinion, which specific domains in HNC patients care are most valuable to assess?	
Domain: Symptoms (*n*, column%)	
Always	63 (60.0)
Quite often	39 (37.1)
Sometimes	2 (1.9)
Rarely	1 (1.0)
Never	0 (0.0)
Missing	28
Domain: Emotional well-being (*n*, column%)	
Always	51 (48.6)
Quite often	41 (39.0)
Sometimes	12 (11.4)
Rarely	1 (1.0)
Never	0 (0.0)
Missing	28
Domain: Social functioning (*n*, column%)	
Always	44 (41.9)
Quite often	46 (43.8)
Sometimes	10 (9.5)
Rarely	5 (4.8)
Never	0 (0.0)
Missing	28
Domain: Financial burden (*n*, column%)	
Always	24 (23.3)
Quite often	30 (29.1)
Sometimes	33 (32.0)
Rarely	12 (11.7)
Never	4 (3.9)
Missing	30
Domain: Other ^1^ (*n*, column%)	4
Do you believe there is any evidence supporting PROMs in HNC patients care in terms of benefits and cost effectiveness?	
I am unsure about the extent of evidence	25 (37.3)
I disagree that there is sufficient evidence	4 (6.0)
Yes, I believe that there is substantial evidence	38 (56.7)
Missing	28
At which phases of HNC treatment are PROMs most valuable? (multiple answers possible; *n*, column%)	
During active treatment	82 (78.8)
During the early stages of follow-up (<2–3 years after the end of treatment)	74 (71.2)
At diagnosis	68 (65.4)
During long-term follow-up (>2–3 years after the end of treatment)	66 (63.5)
At disease progression or recurrence	49 (47.1)
During palliative care (after completion of anticancer therapy)	43 (41.3)
What specific PROMs have you found relevant in HNC patients care? (multiple answers possible; *n*, column%)	
EORTC QLQ-H&N43 ^2^	88 (89.8)
MDADI (MD Anderson Dysphagia Inventory)	51 (52)
FACT-H&N (Functional Assessment of Cancer Therapy-Head and Neck)	40 (40.8)
VHI (Voice Handicap Index)	25 (25.5)
Other ^3^	6 (6.1)

^1^ Impact of symptoms on function, e.g., eating out, engaging in conversation; Carbon foot print impact; Mobility; Taste disturbance; Fatigue; ^2^ (European Organization for Research and Treatment of Cancer Quality of Life Questionnaire-Head and Neck 43). ^3^ Eating Assessment Tool (EAT 10), Sleep Hygiene Index (SHI), Swallowing Outcome After Laryngectomy (SOAL), STOP questionnaire; Hornheider Screening Instrument; Integrated Palliative Outcome Scale (IPOS); MD Anderson Symptom Inventory (MDASI); University of Washington Quality of Life questionnaire (UW-QOL); Xerostomia Inventory; Xerostomia Questionnaire.

**Table 4:** Perceived barriers for PROM integration among non-users versus users.

	Non-Users (*n* = 45)	Users (*n* = 88)	*p* ^1^
System-level barriers			
Lack of reimbursement for using PROMs	16 (35.6)	30 (34.1)	1.000
Lack of appropriate PROMs	16 (35.6)	16 (18.2)	**0.045**
Limited resources or infrastructure at institution	29 (64.4)	38 (43.2)	**0.033**
Limited technical capacity	19 (42.2)	35 (39.8)	0.932
Lack of a “one-size-fits-all” approach	17 (37.8)	34 (38.6)	1.000
Administrative-level barriers			
Legal, liability and/or regulatory concerns	12 (26.7)	22 (25.0)	1.000
Uncertainty on how to assess impact of PROM assessments and quality	15 (33.3)	27 (30.7)	0.909
Lack of shared values (disagreement on purpose and use among stakeholders)	7 (15.6)	22 (25.0)	0.305
Concerns about costs	15 (33.3)	25 (28.4)	0.699
Provider-level barriers			
Lack of healthcare experience and training in interpreting PROM results	28 (62.2)	31 (35.2)	**0.005**
Do not see benefits to using PROMs	6 (13.3)	6 (6.8)	0.357
Concerns about disruptions in workflow caused by PROMs	12 (26.7)	24 (27.3)	1.000
Lack of time	34 (75.6)	48 (54.5)	**0.030**
Technological and logistical challenges	24 (53.3)	36 (40.9)	0.239
Lack of support regarding how to implement and use PROMs	34 (75.6)	40 (45.5)	**0.002**
Patient-level barriers			
Accessibility concerns	13 (28.9)	43 (48.9)	**0.043**
Lack of buy-in (patients consider irrelevant, unsure on data use)	14 (31.1)	33 (37.5)	0.591
Concern about patient burden	26 (57.8)	47 (53.4)	0.768

^1^ *p*-values are based chi-squared tests.

## Data Availability

Data are available upon reasonable request from the corresponding author.

## Supplementary Materials

The following supporting information can be downloaded at: https://www.mdpi.com/article/10.3390/curroncol33050275/s1: EORTC survey; Participants by country of origin.

## Abbreviations

APCOT	App-Controlled Treatment Monitoring and Support Trial
CE	Ethics Committee
COSMIN	COnsensus-based Standards for the selection of health Measurement Instruments
ePROMs	Electronic Patient-Reported Outcome Measures
ePRO	Electronic Patient-Reported Outcome
EORTC	European Organisation for Research and Treatment of Cancer
EORTC QLQ-C30	European Organisation for Research and Treatment of Cancer Quality of Life Questionnaire Core 30
EORTC QLQ-H&N43	European Organisation for Research and Treatment of Cancer Quality of Life Questionnaire Head and Neck 43
EORTC QLQ-BR23	European Organisation for Research and Treatment of Cancer Quality of Life Questionnaire Breast Cancer 23
EORTC QLQ-LC13	European Organisation for Research and Treatment of Cancer Quality of Life Questionnaire Lung Cancer 13
EORTC QLQ-PATSAT-C33	European Organisation for Research and Treatment of Cancer Quality of Life Questionnaire Patient Satisfaction with Cancer Care 33
EORTC QLQ-COMU26	European Organisation for Research and Treatment of Cancer Quality of Life Questionnaire Cancer Outpatient Module 26
EQ-5D-5L	EuroQol 5-Dimension 5-Level questionnaire
ESMO	European Society for Medical Oncology
FACT-H&N	Functional Assessment of Cancer Therapy Head and Neck
FACTG	Functional Assessment of Cancer Therapy General
FACIT	Functional Assessment of Chronic Illness Therapy
HADS	Hospital Anxiety and Depression Scale
HCPs	Healthcare Professionals
HNC	Head and Neck Cancer
HRQOL	Health-Related Quality of Life
IPOS	Integrated Palliative Outcome Scale
MDADI	MD Anderson Dysphagia Inventory
MDASI	MD Anderson Symptom Inventory
PROMIS	Patient-Reported Outcomes Measurement Information System
PROMs	Patient-Reported Outcome Measures
PROs	Patient-Reported Outcomes
PRO-CTCAE	Patient-Reported Outcomes version of the Common Terminology Criteria for Adverse Events
QOL	Quality of Life
QR	Quick Response
SD	Standard Deviation
SF-12	12-item Short Form Health Survey
SF-36	36-item Short Form Health Survey
SHI	Sleep Hygiene Index
SMS	Short Message Service
SOAL	Swallowing Outcome After Laryngectomy
STOP	STOP questionnaire
STROBE	Strengthening the Reporting of Observational Studies in Epidemiology
UW-QOL	University of Washington Quality of Life questionnaire
VHI	Voice Handicap Index
