# Supplementary material for: Integration of Patient-Reported Outcome Measures in Clinical Practice for Head and Neck Cancer Patients: A Cross-Sectional Survey
Source: Curr Oncol. 2026 May 8;33(5):275. doi: 10.3390/curroncol33050275 (PMC13206621; doi:10.3390/curroncol33050275)
Supplement: Supplementary file 1 [file curroncol-33-00275-s001.zip › EORTC PRO HNC survey.pdf]

Survey on the use of patient-reported outcome measures (PROMs) in HNC clinical practice

A survey of the EORTC Head and Neck Young Investigators Group

We kindly invite you to complete and distribute this survey about patient-reported outcome measures (PROMs) in head and neck cancer (HNC) clinical practice.

PROMs are measurement tools, usually questionnaires that directly capture patients' perspectives on their health status, symptoms, and quality of life. In HNC where treatments can affect speech, swallowing, appearance, and overall quality of life, PROMs provide a perspective that enables healthcare professional to assess the effectiveness of interventions from the patient's viewpoint. Supported by a growing body of clinical evidence, many oncological societies, such as European Society for Medical Oncology (ESMO) and American Society of Clinical Oncology (ASCO) recommend using PROMs in cancer care.

In this survey, we would like to learn about your experiences and expectations around PROMs and their use in clinical practice.

Who should complete the survey?

We are looking for all healthcare professionals involved in HNC patients care to complete our survey, regardless of whether they use PROMs and whether they are members of EORTC.

After completing the survey, please kindly distribute it within HNC societies, organizations or professionals caring for HNC patients.

Thank you for taking part!

About the survey

In this survey, we would like to learn about your experiences and expectations around PROMs and their use in clinical practice. The survey has been approved by the ethics committee of the Instutut Jules Bordet, Université Libre de Bruxelles (CE 3749/202401111)

The survey consists in two sections, general and specific to HNC disease and will take about 20 to 25 minutes to complete.

Who should complete the survey?

We are looking for **all healthcare professionals involved in HNC patients care** to complete our survey, regardless of whether they do or do not use PROMs.

Thank you for taking part!

The EORTC Head and Neck Young Investigators Group

If you have any questions, you may contact

Dr. Tatiana Dragan tatiana.dragan@hubruxelles.be, member of the QLQ EORTC and HNC Y-ECI EORTC groups

Dr. Petr Szturz (szturz@gmail.com, EORTC Y-ECI Board representative, chair of the H&N cancer Y-ECI group)
Jens Lehmann, PhD (jens.lehmann@i-med.ac.at, chair of the QLG Y-ECI group)

Demographics

1. Please enter your email address below _____________________________________
   Privacy Note: Your email address will only be used for research purposes and will not be shared with any third parties.

2. In what country do you work?

   [country selection]

3. Are you a member of any of the EORTC groups?
   Yes
   No
4. To which gender identity do you most identify?
   Woman
   Man
   Non-binary
   I prefer not to disclose
   I use a different term (specify)

5. To which age range do you belong?
   >18 - 29 years
   30 - 39 years
   40 - 49 years
   50 - 59 years
   60 - 69 years
   => 70 years
6. What is your profession?
   Physician (any medical specialty)
   Study/research nurse (e.g. nurse dedicated to work with clinical trials)
   Nurse
   Supportive care professional (physiotherapist, nutritionist,...)
   Other, please specify: ____________
7. How long have you been working with HNC (including your training period)?
   Less than one year

1 - 3 years
4 - 10 years
11 - 20 years
More than 20 years

8. In what type of healthcare facility do you practice? (tick all that apply)
   Academic/university hospital
   Regional hospital, serving a geographic region
   County hospital, acute with less than 200 beds
   Tertiary referral hospital
   Private practice
   Ambulatory care
   Other, please specify ____________
9. How many new HNC patients do you see per month in your practice
   1-5
   6-15
   >15
10. How much of your work time (in %) on average, do you spend per week seeing HNC patients?

Use of patient-reported outcome measures (PROMs) in clinical practice

11. Do you use PROMs in your ***clinical practice***? (i.e., outside clinical trials)
   Yes – almost daily
   Yes – at least a couple of times per month
   Yes – at least a couple of times per month
   Yes – but only occasionally (i.e., very infrequently)
   No, never
12. How do patients complete PROMs in your clinical practice? (i.e., outside clinical trials)
   Paper questionnaires
   Electronic questionnaire assessment
   Both formats
13. Are you aware of other healthcare professionals at your institution using PROMs in clinical practice?
   Yes, people at my department
   Yes, people at other departments
   Yes, people both at my department and other departments
   No, not that I am aware of

FOR PROM NON-USERS: (item 9, responded “no, never”):

The following list presents potential reasons for not using PROMs in clinical practice. **Please rate for each of the reasons if this is a reason for you to not use PROMs in clinical practice.**
(Yes / No / I do not know)

Accessibility concerns (patients not able to complete)
Lack of buy-in (patients consider irrelevant, unsure on data use)
Concern about patient burden (too complicated time-consuming)
Lack of healthcare experience and training in interpreting PROM results
I don’t see any benefit to using PROMs
Concerns about disruptions in workflow caused by PROMs
Lack of time for PROMs during clinical interactions
Technological and logistical challenges (difficulty finding PRO system, too complex)
Lack of support regarding how to implement and use PROMs (e.g. management support)
Concerns about costs for setting up PRO Systems
Lack of shared values (disagreement on purpose and use among stakeholders)
Uncertainty on how to assess impact of PROM assessments and quality
Legal, liability and/or regulatory concerns around PROMs (unclear on responsibility for review on reported symptoms, lack of framework for PROM assessment, privacy concerns,...)
Lack of a “one-size-fits-all” approach (lack of a single PRO system across different settings)
Limited technical capacity of PRO system (system not sufficiently integrated)
Limited resources or infrastructure at institution (no in-house expertise)
Lack of appropriate PROMs to use (no appropriate questionnaires)
Lack of reimbursement for using PROMs or reviewing PROM results
Other, please specify ____________

14. How long have you been using PROMs in clinical practice? *(conditional logic dependent on responses to item 9)*

Less than 1 year
1 -3 years
4 - 5 years
6 -10 years

More than 10 years

15. Who introduces the PROMs to the patient? (tick all that apply)
    Nurses
    Administrative staff
    PRO coordinator (i.e., a person dedicated to PROM assessment)
    I do not know
    Physicians
    Other, please specify ____________

16. Where do patients complete PROMs ?
    On-site at your institution
    Remotely at home
    Combination of above

17. How do you ensure that patients participate in the remote assessments? (Tick all that apply)
    Automated reminders (SMS, email, app, …)
    Offering a support hotline or contact information
    Optimizing the timing of assessments
    Training patients on when and how to complete questionnaires
    Calling/following-up non-adherent patients
    We do not use strategies to ensure participation
    Other, please specify ____________

18. What percentage of your patients is invited to complete PROM at least once during the course of their treatment (approximatively)?

(slider 0 to 100)

19. At what time points during the treatment trajectory do you use PROMs? (tick all that apply)
    At diagnosis
    During active treatment
    At disease progression or recurrence
    During the early stages of follow-up (< 2-3 years after the end of treatment)
    During long-term follow-up (> 2-3 years after the end of treatment)
    During palliative care (after completion of anticancer therapy)
    Other, please specify ____________

20. What PROMs do you use? (Tick all that apply)
    EORTC QLQ-C30 (European Organisation for Research and Treatment of Cancer - Quality of Life Questionnaire)
    EORTC disease-specific questionnaires (e.g., EORTC QLQ-HN43.)

Other EORTC questionnaires (EORTC QLQ-PATSAT, QLQ-COMU26, …)
EQ-5D
SF-36 (36-Item Short Form Health Survey)
PRO-CTCAE (Patient-Reported Outcomes version of the Common Terminology Criteria for Adverse Events)
HADS (Hospital Anxiety and Depression Scale)
FACIT questionnaires (Functional Assessment of Chronic Illness Therapy) like the FACT-G
PROMIS (Patient-reported Outcomes Measurement Information System) like the PROMIS-29
We have developed our own questionnaire for our patients

I do not know

Other, please specify ____________

21. What do you use PROMs for in your clinical practice? **Never/Rarely/Sometime/Quite often/Always**

Monitoring patients' general health status
Monitoring mental health problems
Monitoring the response to treatment
Diagnostic and support for screening (e.g. dietary screening or psycho-oncological screening)
To inform treatment decisions (based on PROM results)
To inform and guide the choice of aftercare based on PROM results (eg specialist oncology care , GP-led care, nurse-led care, self-management)
To support communication between patients and professionals
To support communication within the healthcare team
For other purposes, please specify_________________

22. The following list presents potential barriers and concerns that you may have encountered in the past when implementing PROMs in clinical practice.

Please rate for each of the reasons if this was relevant barriers to overcome for your PROM implementation: **Yes/No/I do not know**

Accessibility concerns (patients not able to complete)
Lack of buy-in (patients consider irrelevant, unsure on data use)
Concern about patient burden (too complicated time-consuming)

Lack of healthcare experience and training in interpreting PROM results
I don't see any benefit to using PROMs
Concerns about disruptions in workflow caused by PROMs
Lack of time for PROMs during clinical interactions

Technological and logistical challenges (difficulty finding PRO system, too complex)
Lack of support regarding how to implement and use PROMs (e.g. management support)
Concerns about costs for setting up PRO Systems
Lack of shared values (disagreement on purpose and use among stakeholders)
Uncertainty on how to assess impact of PROM assessments and quality
Legal, liability and/or regulatory concerns around PROMs (unclear on responsibility for review on reported symptoms, lack of framework for PROM assessment, privacy concerns,...)
Lack of a "one-size-fits-all" approach (lack of a single PRO system across different settings)
Limited technical capacity of PRO systems (systems not sufficiently integrated)
Limited resources or infrastructure at institution (no in-house expertise)
Lack of appropriate PROMs to use (no appropriate questionnaires)
Lack of reimbursement for using PROMs or reviewing PROM results
Other, please specify ____________

Use of patient-reported outcome measures (PROMs) in clinical tials

The following questions concern the use of PROMs in clinical trials

23. Have you ever used PROMs in clinical trials?

Yes, I use PROMs all the time
Yes, I use PROMs quite often
Yes, I sometimes use PROMs
Yes, but I rarely use PROMs
No, I never use PROMs

24. How were PROMs administered to patients in those trials/studies?

Paper questionnaires
Electronic assessment
Both formats
Not applicable, never used PROMs in clinical trials

25. If you were using PROMs as part of a hypothetical trial or study, how would you prefer to administer them to patients?"

Paper questionnaires
Electronic assessment

Both formats

Use of PROMs in head and neck cancer patients care

26. In your opinion, which specific domains in HNC patients care, are most valuable to assess using PROMs: **Never/Rarely/Sometime/Quite often/Always**
    Symptoms (e.g. pain, swallowing problems, xerostomia etc)
    Emotional well-being, body image and self-esteem
    Social functioning, familial roles, work, caregiving responsibilities
    Financial burden
    Other, please specify ____________

27. What specific PROMs have you found relevant in HNC patients care
    EORTC QLQ-H&N43 (European Organization for Research and Treatment of Cancer Quality of Life Questionnaire - Head and Neck 43)
    MDADI (M. D. Anderson Dysphagia Inventory)
    FACT-H&N (Functional Assessment of Cancer Therapy - Head and Neck):
    VHI (Voice Handicap Index)
    Other, please specify______________

28. Do you believe there is any evidence, according to the scientific literature, supporting PROMs in HNC patients care in terms of benefits and cost-effectiveness?
    Yes, I believe that is a substantial evidence
    I am unsure about the extent of evidence
    I disagree that there is sufficient evidence

29. In your opinion, at which phases of HNC treatment are PROMs most valuable? (tick all that apply)
    At diagnosis
    During active treatment
    At disease progression or recurrence
    During the early stages of follow-up (< 2-3 years after the end of treatment)
    During long-term follow-up (> 2-3 years after the end of treatment)
    During palliative care (after completion of anticancer therapy)
    Other, please specify ____________

30. Based on your experiences, what suggestions do you have for improving the integration and utilization of PROMs in HNC patients care clinical practice?_____________________________
