# Supplementary figures and images for: Integration of Patient-Reported Outcome Measures in Clinical Practice for Head and Neck Cancer Patients: A Cross-Sectional Survey
Source: Curr Oncol. 2026 May 8;33(5):275. doi: 10.3390/curroncol33050275 (PMC13206621; doi:10.3390/curroncol33050275)

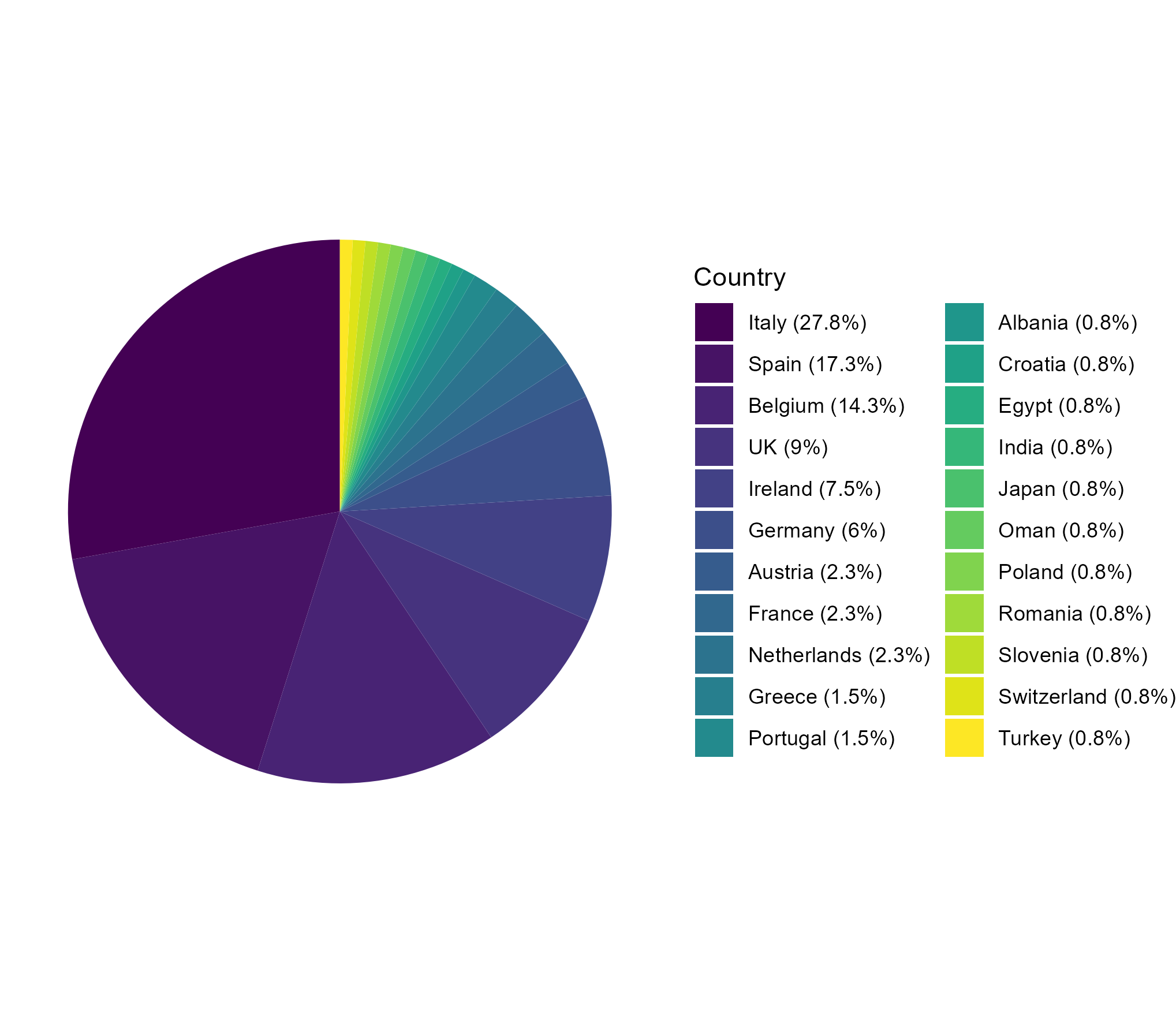

Supplement: Supplementary file 1 [file curroncol-33-00275-s001.zip › Figure Participants by country of origin.png]
